# Beta Diversity of Plant-Pollinator Networks and the Spatial Turnover of Pairwise Interactions

**DOI:** 10.1371/journal.pone.0112903

**Published:** 2014-11-10

**Authors:** Daniel W. Carstensen, Malena Sabatino, Kristian Trøjelsgaard, Leonor Patricia C. Morellato

**Affiliations:** 1 Departamento de Botânica, Laboratório de Fenologia, Plant Phenology and Seed Dispersal Group, Universidade Estadual Paulista (UNESP), Rio Claro, São Paulo, Brazil; 2 Laboratorio Ecotono-CRUB, Universidad Nacional del Comahue & CONICET, San Carlos de Bariloche, Río Negro, Argentina; 3 Department of Bioscience, Aarhus University, Aarhus, Denmark; 4 Department of Biotechnology, Chemistry and Environmental Engineering, Aalborg University, Aalborg, Denmark; Central China Normal University, China

## Abstract

Interactions between species form complex networks that vary across space and time. Even without spatial or temporal constraints mutualistic pairwise interactions may vary, or rewire, across space but this variability is not well understood. Here, we quantify the beta diversity of species and interactions and test factors influencing the probability of turnover of pairwise interactions across space. We ask: 1) whether beta diversity of plants, pollinators, and interactions follow a similar trend across space, and 2) which interaction properties and site characteristics are related to the probability of turnover of pairwise interactions. Geographical distance was positively correlated with plant and interaction beta diversity. We find that locally frequent interactions are more consistent across space and that local flower abundance is important for the realization of pairwise interactions. While the identity of pairwise interactions is highly variable across space, some species-pairs form interactions that are locally frequent and spatially consistent. Such interactions represent cornerstones of interacting communities and deserve special attention from ecologists and conservation planners alike.

## Introduction

Spatial turnover of diversity, or beta diversity, has long been recognized as an important part of species diversity [1]–[3]. The beta diversity of a region is high if local sites within the region have unique species compositions so that no single site samples the majority of the total regional diversity. Beta diversity is fundamental to many aspects of diversity in ecological communities and in conservation planning, e.g. when determining the number of protected areas required to achieve biodiversity representation [4]–[6].

Interactions between species are an important, but often ignored, part of biodiversity [7]. Complete diversity assessments, and questions on drivers of diversity, should refer to both species and interactions, but this is still rarely done. Recent studies indicate that we cannot make solid inferences about regional interaction diversity solely from information about species diversity. For instance, Burkle and Alarcón [8] showed that community dissimilarity of plant and pollinator species was highly predictable along an environmental gradient whereas the dissimilarity of the interactions between them was poorly explained. Poisot et al. [9] similarly found no correlation between beta diversity of species and interactions for host-parasite networks indicating that species and interactions are sorted through different mechanisms. Sabatino et al. [10] demonstrated that interaction richness increases twice as fast as species richness with increasing area. Furthermore, interactions between specialists have been shown to be the most vulnerable to habitat fragmentation, while interactions between the core of generalists are more robust [11]. Still, our knowledge of the regional dynamics of interaction diversity, and the relationship between diversity of interactions and species, is in its infancy. Increased knowledge of the drivers of beta diversity of interactions is important to community ecology as it may illuminate what determines the identity of pairwise interactions and whether they are predictable from the composition of species, but it may also guide conservation planning by aiding the understanding of ecosystem functioning and interaction-based ecosystem services [12].

Mutualistic networks consist of two interacting communities and, consequently, their interactions are often analysed using a bipartite network approach [13]. Such networks consist of two types of nodes, e.g. plants and pollinators, connected by links. Detailed structures of pollination networks, such as species degree, core composition, and the identity of pairwise interactions, are highly dynamic over time [8], [14]–[20]. This variation is caused by temporal differences in species composition and phenology [15]–[18], [21] but also by a strong lability in the identity of pairwise species interactions, i.e. interaction rewiring [18], [22]. Interactions are temporally constrained if phenologies of potentially interacting species are decoupled, but they may also be spatially constrained [23]. One obvious reason is the turnover in species composition, i.e. spatial species-driven interaction turnover. However, a pair of species interacting in one area might be present in another area without interacting, i.e. spatial interaction rewiring (see [19] for a similar distinction in studies of temporal interaction turnover).

Poisot et al. [9] propose that the overall dissimilarity in interactions between networks is the sum of the dissimilarities caused by species turnover and interaction turnover (i.e. interaction rewiring). In the current study, we focus on the interaction turnover component. This variability in species interactions is not well studied but recent progress has identified potential drivers. First, species abundance affects the probability of interactions [24]. Neutral theory states that individuals interact randomly and that species interact with a probability determined by their abundance product [25]. Because population densities determine the probabililty of pairwise species encounters, relative abundances ultimately determine the realization of pairwise interactions. Second, when species meet, trait matching will constrain or promote the realization of pairwise interactions [23], [26]. Compatibility of traits between species can be viewed quantitatively rather than purely qualitatively, and species with highly matching traits will likely interact with higher probability than species with poorly matching traits. Traits may also vary within-species across populations, increasing interaction turnover across space [27]. Finally, the local realization of pairwise interactions might be affected by competitive or facilitative effects from other species or interactions. Such mechanisms will potentially create complex community effects which are not easily tested. Network structure is determined by the combined effects of neutrality and trait matching [21], [28] but as drivers of interaction turnover they have not been properly tested.

**Figure 1:**
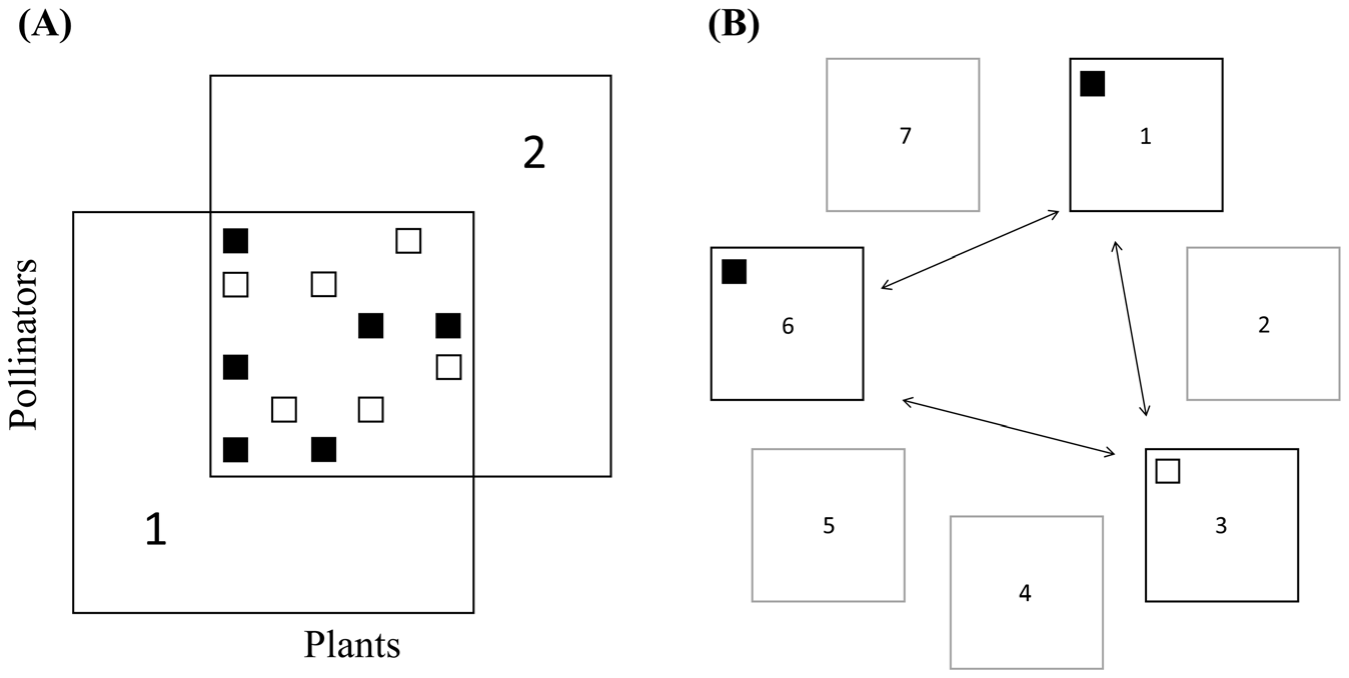
Site-pair comparison and interaction specific site-pair combinations. A) Site-pair comparison. Site 1 and 2 each have unique plants and pollinators. The central square represents the interaction-matrix between shared species. Here, six interactions are present in both sites (interaction consistency, filled squares) and six interactions are only observed in one of the two sites (interaction turnover, open squares). Unique species to either site 1 or 2 were discarded and only the central matrix was used for analysing the turnover of pairwise interactions. B) Interaction specific site-pair combinations. This (hypothetical) interaction is observed at sites 1 and 6 (filled squares) while the species pair is also present at site 3, however without interacting (open square). One or both species are absent from the remaining sites (in grey) and they are excluded from the analysis for this particular interaction. Three site-pair combinations are possible in this case; 1↔3 and 6↔3: interaction turnover and 1↔6: no interaction turnover (interaction consistency).

Here, we quantify the spatial turnover in plant-pollinator interaction networks by examining the beta diversity of species and interactions between network pairs across seven sites. Then, we restrict the analysis to the shared networks between sites (i.e. only including shared species, see Figure 1A) and test the effect of interaction properties and site characteristics upon interaction turnover. Specifically we ask: 1) to what extent beta diversity of plant species, pollinator species, and interactions follow similar trends across space, and 2) which interaction properties and site characteristics are related to the probability of turnover of pairwise interactions across space. That is, when looking at each specific interaction between species pairs shared between two or more sites, can we then determine which interactions are more likely to turn over and under which conditions?

On larger spatial scales, increasing dissimilarity between communities with increasing geographical distance, i.e. distance decay, is a well-documented pattern with respect to species composition [29] and has been shown once also for food webs [30]. The pattern, however, seems to become less clear at smaller spatial scales (1–3 km) and is thus far poorly explored with respect to interactions (but see [8]). Although Burkle and Alarcón [8] found no correlation between distance and species and interaction similarity across pollination networks, Dáttilo et al. [31] found a decreasing similarity in ant and plant composition with increasing distance. Thus, we expect a positive correlation between geographical distance and turnover of species. While species and interactions could be sorted through different mechanism [9] we also expect a positive correlation between geographical distance and interaction turnover, although the predictability may be lower.

For our second question we focus on two interaction properties: average interaction frequency and interaction generalization, and three site characteristics: local flower abundance, local network species richness, and geographical distance between sites. Average interaction frequency measures the average frequency of a given pairwise interaction when both species are present. We argue that this is a good proxy for trait complementarity and behavioural preferences between species pairs. Lacking detailed information on species-specific traits, and how they would combine in the given system, interaction frequency is likely an outcome of such mechanisms [27]. We expect average interaction frequency to be negatively correlated with the probability of turnover. Interaction generalization is a measure of the generalization level of the species pair forming the interaction in question. Ecological specialization, i.e. the use of a relatively small proportion of the available interaction partners [32], [33], is likely connected to less promiscuity, and thus a higher consistency of interactions between more specialized species can be expected [34], [35]. Flower abundance has repeatedly been shown to be important to determine network structure [21], [24], [36]–[40], and we expect a change in local flower abundance between sites to influence interaction turnover so that a decrease in flower abundance of a given plant species will lower the probability that pairwise interactions, involving the same plant species, are realized. Finally, as explained above, different mechanisms of interference from other species might promote interaction turnover. We therefore expect that species richness of a given site can affect the realization of pairwise interactions by altering the competitive or facilitative context. Predicting the direction of such an effect is problematic as it is likely highly system-specific. However, recent experimental work on plant-pollinator systems indicates that an increase in species richness could increase the probability of turnover of interactions [41].

We show a positive correlation between geographical distance and beta diversity of species and interactions. Our findings indicate that the identity of pairwise interactions is highly variable across space, but that local flower abundance is important for the realization of interactions. Furthermore, those pairwise interactions that are locally frequent will also tend to be consistent across space if no temporal or spatial constraints are imposed on the species. These interactions could be of key importance for species in obligate or facultative mutualisms and form consistent elements in otherwise highly variable interaction networks.

## Materials and Methods

### Study site

We conducted our study in the National Park of Serra do Cipó and its buffer zone, an Environmental Protection Area, Morro da Pedreira. This protected area, addressed together as Serra do Cipó, is located in the southern end of the Espinaço mountain chain, in the state of Minas Gerais, SE Brazil. Here, *campos rupestres*, or rupestrian fields, is a common habitat type between 1000–1400 m a.s.l. It is characterized by a species-rich vegetation of mostly small sclerophyllous evergreen shrubs and herbs associated with rocky outcrops and quartzitic or sandy soils with high aluminium and low nutrient contents [42], [43], [44]. Large variations in daily temperature, strong winds, frequent fires, and little accessible water during the dry season are important stressors, and a xeromorphic, fire-resistant vegetation has evolved [45]. *Campos rupestres* form isolated hills of sandstone and quartzite emerging as a mosaic surrounded by cerrado, forest, or caatinga. This mosaic landscape structure may be an important reason for *campos rupestres* having one of the highest levels of floristic endemism found in the cerrado biome [41], [46].

Within Serra do Cipó, we chose seven sites of *campos rupestres*. Sites were chosen so as to minimize abiotic differences between them in order to reduce environmental noise and restrict the focus towards biotic changes across sites. All sites were within an altitudinal range of 1073–1260 m a.s.l. with similar wind exposition, soil substrate, and floral species richness (see File S1). All field activities were authorized by ICMBio of the Brazilian ministry of environment.

### Observation of plant-pollinator interactions

Sampling was done in 2012 during the peak flowering season (October–December) [47]. Intensive sampling restricted to the peak flowering season reduced the introduction of forbidden links due to phenological mismatches [23]. At each site we sampled ten 1 m^2^ plots along a 200 m long curvilinear transect. These plots were placed in a manner that maximized the number of observed plant species. We sampled one site per day with a fixed weekly rotation among sites. At each plot, we made a 15-min census observing all flowering plant species for flower visitors. We only registered animals touching reproductive floral parts and they are here operationally defined as pollinators. Daily census was done between 9 and 14 hours, covering the main activity period of the pollinators [47]. Observations were done every day by DWC and MS, except when raining. Sampling accumulated to six days per site and 252 observation hours over 44 days, resulting in a total of 2271 observed interactions. Each site was sampled 36±1 h in total, and all sites are regarded as equally sampled (File S2). At each plot, flower abundance was estimated for each species by counting the number of individuals and flowers within each station. Pollinators were collected when identification in the field was not feasible. Specimens of the observed plant species were likewise collected. Species were subsequently identified with the aid of a reference collection and several specialists (see acknowledgements). Voucher specimens of plants and pollinators are deposited at Herbário Rioclarense, UNESP, Rio Claro (HRCB), Universidade Federal de Minas Gerais (UFMG), and Coleção Entomológica Padre Jesus Santiago Moure, UFPR (DZUP), Brazil.

### Data analysis

For each site, we constructed an interaction matrix with pollinator species in rows and plant species in columns. Interaction frequency for each interacting species pair was the number of interactions per flower per 15 min of observation time. We standardized interaction frequency per flower in order to account for differences in flower abundance of observed individuals. Using these matrices, a quantitative bipartite plant-pollinator network was constructed for each site. For quantification of beta diversity and turnover probability, we used only the presence-absence of interactions as a clear distinction of turnover vs. no turnover was necessary. The quantitative measures (interaction frequencies) were used only as an explanatory variable in the model (see below).

We analysed the networks in two steps. First, we calculated the beta diversity of species, of interactions between shared species, and of all interactions in the network, between all pairwise combinations of the seven sites, a total of 21 site-pair combinations. All measures of network beta diversity were calculated using the framework proposed by Poisot et al. [9]. Numerous variations of beta diversity measures exist, with little consensus on which measure is most appropriate in a given situation [2]. Here, we applied a beta diversity measure widely used in ecology, β_w_ [1]. We applied this “broad sense” measure (*sensu* [2]) as it incorporates both differences in richness and composition between sites. It is defined as: 
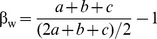
, where *b* is the number of items unique to the first site, *c* is the number of items unique to the second site, and *a* is the number of shared items between two sites. When calculating beta diversity of interactions between shared species (β_OS_) *b* and *c* represent interactions (among the shared species) found only in one of the networks, and *a* represents interactions in common between sites (see Figure 1A). This measure thus explicitly defines the spatial interaction rewiring as it ignores the part of network turnover that is caused by species turnover. Total interaction beta diversity (β_WN_) was calculated using all the interactions in the network, including those between species that are unique to one or the other site. Species beta diversity was calculated for plant species alone (β_Plants_), pollinator species alone (β_Pollinators_), and for plants and pollinators together (β_Species_). We calculated mean beta diversity values by averaging the relevant beta diversity for each of the 21 site-pair combinations. The R package *betalink* [9] was used to calculate all measures of beta diversity.

Secondly, we isolated the common networks of site-pairs, that is, we included only those species present at both sites, and then extracted each interaction within this “common network” (Figure 1A). For each of these interactions, we tested two interaction properties (average interaction frequency and interaction generalization) and three site characteristics (difference in flower abundance per plant species, difference in network species richness, and the geographical distance between sites) and their relation to the binary response variable of whether the interaction would turnover or not between site-pairs. Thus, for each species pair that occurred in at least two sites, and interacted in at least one of these, we obtained at least one event of interaction turnover or interaction consistency. For each unique interaction between a plant and a pollinator species we got one or more site-pair combinations. Each data entry is therefore an interaction specific site-pair combination (Figure 1B). We used a binomial model with a stepwise selection procedure to test the effect of the interaction properties and site characteristics on the turnover of the interaction for each interaction specific site-pair combination.

Average interaction frequency is the average of how frequently a given pairwise interaction is observed when both species are present at a site. This measure is therefore a property of the given interaction and each unique interaction of the entire system (the metaweb, *sensu* [48]) thus has an average interaction frequency. It was calculated as the sum of all the interaction frequencies registered between two particular species (i.e. the interaction frequency in the metaweb) divided by the number of sites where both species occur (interacting or not). Interaction generalization is a measure of the generalization level of the species pair forming the interaction in question. It was calculated by averaging the total number of different interacting partners of the two species across all sites (i.e. the mean of the degree in the metaweb of the plant and pollinator species). In mutualistic networks, the degree of a species is partly explained by its abundance [28], [37], [39], [40]. We thus used the residual variance from the abundance/degree correlation as species degree for calculating interaction generalization. For a given interaction specific site-pair combination we calculated the difference in flower abundance between sites by subtracting the flower abundance of the one site from that of the other. This was then standardized by the total number of flowers for both sites for that species in order to get a measure of the relative change in flower abundance. This relative difference was used as an explanatory variable in the model. In order to investigate the direction of the effect, that is, whether an increasing abundance would have a positive or negative effect on whether a given interaction would be realized, we subsequently divided the data into those situations where abundance increased between sites and those where abundance decreased between sites. In the model, we further used a standardized difference in species richness between sites, that is, the difference in number of species divided by the total number of species in both sites. Geographical distance was the Euclidean distance between a site-pair. Analyses were implemented in R v. 3.0.1 [49].

## Results

### Network beta diversity

Mean species beta diversity between sites was β_Species_  = 0.59, β_Plants_  = 0.66, and β_Pollinators_  = 0.57 (see File S3 for details). The mean beta diversity of interactions between shared species was β_OS_  = 0.62. Of the 101 plant species observed in total across all seven sites, 65 species were unique to a single site. Of the 199 pollinator species, 83 were unique to a single site.

**Figure 2:**
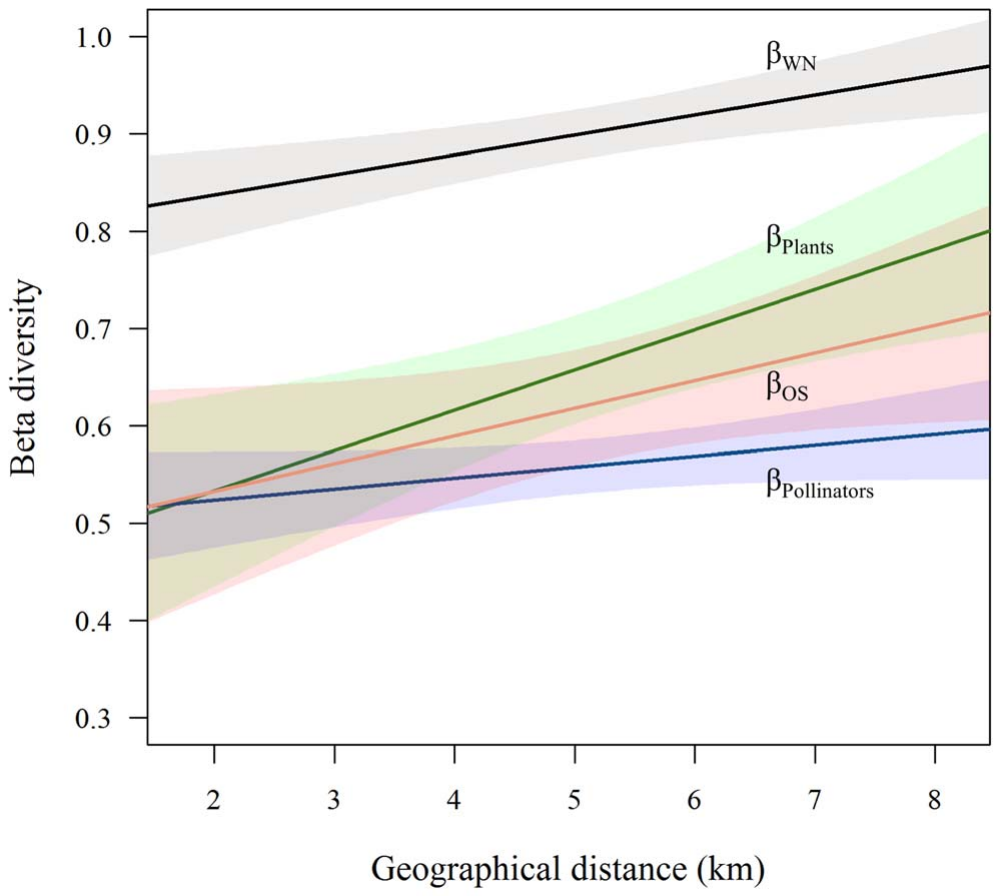
Beta diversity of species and interactions and geographical distance. Total interaction beta diversity β_WN_ (black), plant beta diversity β_Plants_ (green), beta diversity of interactions between shared species β_OS_ (red), and pollinator beta diversity β_Pollinators_ (blue) as a function of geographical distance between sites. All measures relate positively to geographical distance. Only for β_Pollinators_ the correlation was non-significant (β_WN_: P = 0.002, *R* ^2^ = 0.40, *α* = 0.02; β_OS_: P = 0.047, *R* ^2^ = 0.19, *α* = 0.03; β_Plants_: P = 0.004, *R* ^2^ = 0.37, *α* = 0.04; β_Pollinators_: P = 0.086, *R* ^2^ = 0.15, *α* = 0.01). Shaded areas delimit corresponding 95% confidence intervals.

Geographical distance was positively correlated with total interaction beta diversity (β_WN_) (P = 0.002, *R* ^2^ = 0.40, *α* = 0.02, Figure 2), beta diversity of interactions between shared species (β_OS_) (P = 0.047, *R* ^2^ = 0.19, *α* = 0.03, Figure 2), and β_Plants_ (P = 0.004, *R* ^2^ = 0.37, *α* = 0.04, Figure 2) across sites. β_Pollinators_ also showed a positive, albeit not significant, correlation with geographical distance (P = 0.086, *R* ^2^ = 0.15, *α* = 0.01, Figure 2).

### Turnover of pairwise interactions

**Figure 3:**
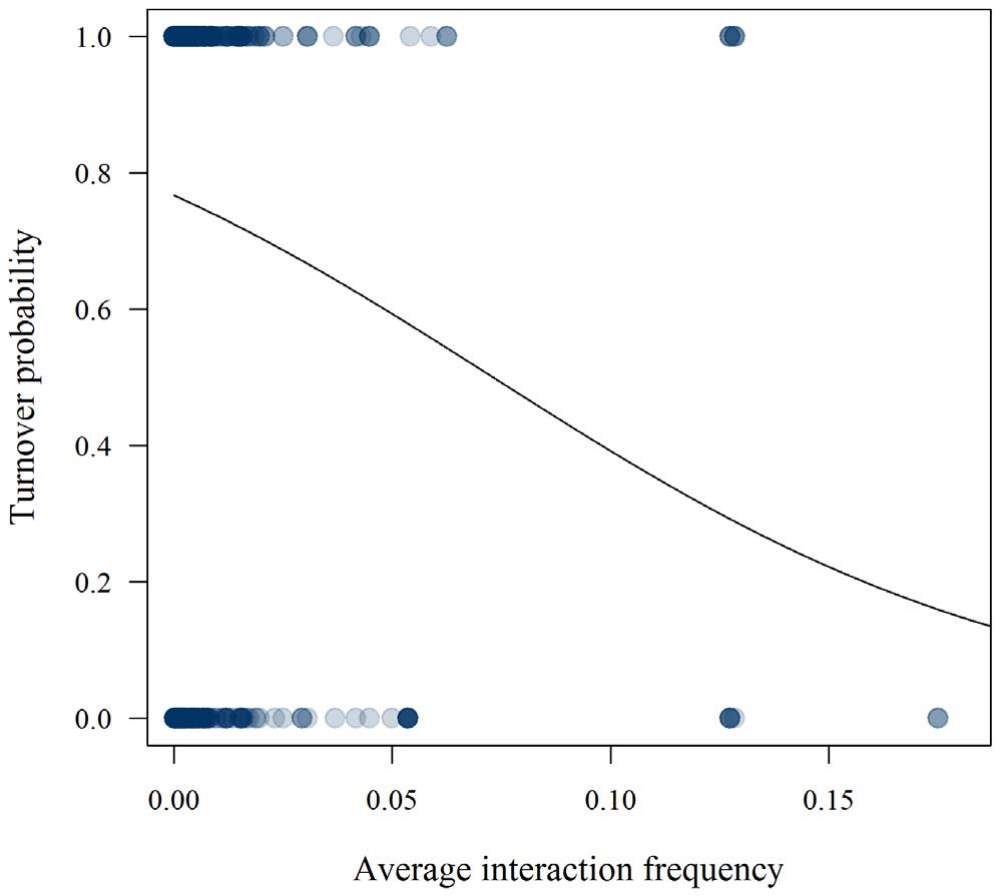
Interaction frequency and interaction turnover. Average interaction frequency is negatively related to the probability of interaction turnover. The more frequent interactions show lower probabilities of turnover between sites. Superimposed points result in darker marks.

**Figure 4:**
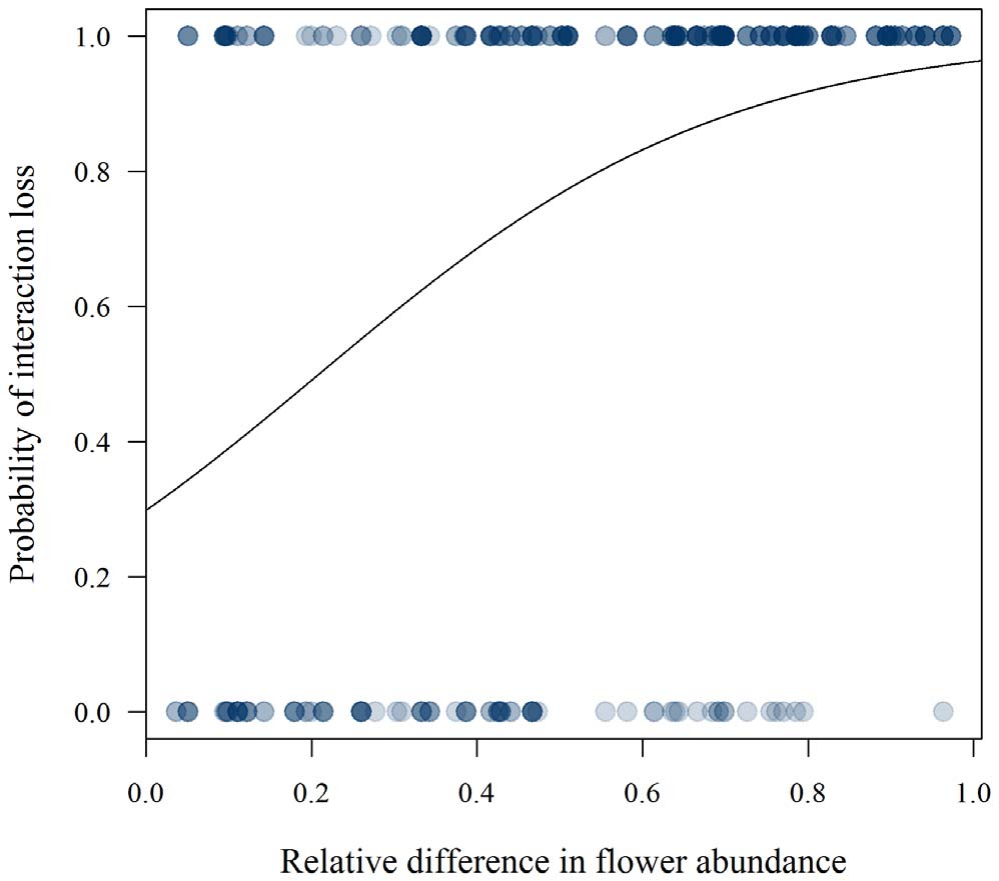
Flower abundance and the probability of losing an interaction. Whether pairwise interactions are realized or not is highly dependent on local flower abundance of the plant species. The figure shows the increasing probability of losing an interaction when going from a site with higher local flower abundance of the plant species to a site with lower local flower abundance. The larger the difference in local flower abundance, the stronger the effect. Superimposed points result in darker marks.

We analysed 1063 interaction specific site-pair combinations. Of these, only 271 (25.5%) were consistent between sites. In the binomial regression analysis average interaction frequency, difference in flower abundance, and geographical distance were included in the best-fit model (Table 1). The effect of average interaction frequency on interaction turnover was negative (Table 1, Figure 3), while difference in flower abundance and geographical distance were positively correlated with interaction turnover (Table 1). The probability of losing an interaction from one site to another increased if flower abundance decreased; the larger the difference in abundance, the stronger the effect (Figure 4). Network species richness and interaction generalization were not included in the best-fit model (Table 1).

**Table 1:** Model terms and their estimates.

	ΔAIC	Terms in model	Coeff.	SE	z value
**Best-fit model**	0				
		average interaction frequency***	−15.34	3.61	−4.24
		flower abundance***	1.14	0.26	4.47
		geographical distance**	0.10	0.04	2.61
**Full model**	2.6				
		average interaction frequency ***	−15.40	3.57	−4.31
		flower abundance***	1.10	0.26	4.32
		geographical distance**	0.10	0.04	2.67
		interaction generalization^NS^	0.17	0.25	0.67
		average interaction frequency: interaction generalization^NS^	11.69	11.96	0.98
		network species richness^NS^	1.22	1.05	1.18

Including standard errors (SE) and z values for the full and best-fit models.

***: P<0.001, **: P<0.01, *: P<0.05, ^NS^: P>0.1.

## Discussion

Pairwise interactions, the fundamental component of complex ecological networks, have proven difficult to predict [8], [50]. We find that pairwise interactions are indeed highly variable across space, but that some types of interactions are more predictable than others. Pairwise interactions that are locally frequent are more consistent across space. We further show that the probability, for a pairwise interaction to be realized, is increased with the local abundance of the plant species forming the interaction. Finally, increased geographical distance between sites significantly increase beta diversity of plant species and interactions and the probability of turnover of pairwise interactions.

As expected, interactions that were locally more frequent showed a lower turnover across sites. Such interactions can be interpreted as linking species of high mutual affinity; if no spatial or temporal constraints are imposed, these species pairs will likely interact, and likely with a high frequency. Such strong interactions are of principal importance in plant reproduction locally [38]. Our results demonstrate their regional importance as interactions that link sites across *campos rupestres* landscape. These interactions represent spatially consistent elements across interaction networks. Hyperdominant species represent a defining set for a biome or a region and will account for a large proportion of the processes within a given system [51]. Understanding this small fragment of the existing diversity will thus greatly increase the understanding of the system. We have here taken a first step towards identifying a set of interactions that in a similar manner is defining for a region.

While the effect of abundance on nestedness, asymmetry, species degree, and other network properties in plant-pollinator networks is well documented [21], [28], [37], [38], the effect of flower abundance on the realization of pairwise interactions is hitherto poorly tested. We show that the probability for a given pollinator to interact with a given plant species depends upon the resource level that the plant is offering at a given site. Flower abundance affects pairwise interactions by increasing the attractiveness of plants with many flowers (i.e. increased resource levels), and hereby influencing behavioural decisions by pollinators. Additionally, abundance affects species' encounter probabilities and such neutral factors have earlier been shown to influence interaction patterns [24], [39]. Interaction strength is directly affected by relative abundances [24] but as we have shown here, interactions may also be entirely lost or gained over space as a function of varying abundances. Negative difference in abundance increases the probability of interaction loss by introducing “neutral forbidden links” [52]. That is, because of low abundances, co-occurring rare species might be constrained from interacting, in spite of otherwise complementing traits. In a strict neutral approach, species identities do not matter – relative abundances alone determine interaction probability [25]. In reality, encounters may be stochastic, but certain species will be more likely to interact if they meet. The strong affinities between certain pairs of species, here indicated by the negative relationship between average interaction frequency and turnover probability, are likely a function of trait complementarity.

Beta diversity of interactions and species were related to geographical distance in a similar positive manner. Plant species not only showed overall higher beta diversity than pollinators; plant beta diversity, contrary to that of pollinators, was significantly correlated with geographical distance. Pollinators are mobile and, all else being equal, will show higher dispersal capabilities in ecological time possibly explaining the lower, and less distance-dependent, beta diversity of pollinator species compared to plants. A similar pattern has been shown for herbivores and their plant hosts [30]. Beta diversity of interactions between shared species (β_OS_) also showed a significant correlation with geographical distance, suggesting that species in neighboring sites are more likely to display similar interaction behaviors compared to species from distant sites. Such a significant relationship has, to our knowledge, not been found earlier. Geographical distance also had a significant effect on the probability of turnover in our model, reaffirming the correlation between β_OS_ and geographical distance. It should be noted, however, that in both cases correlations were significant, but not strongly so. Thus, geographical distance alone explains little of the variation in interactions across space. A combination of subtle variation in community and landscape properties which are spatially autocorrelated could be the cause of the distance effect on interaction turnover.

Interaction generalization (i.e. the mean generalization level of the species involved in a particular interaction) showed no significant effect on the probability of turnover of pairwise interactions. Species with few local interaction partners might indeed appear as specialists, however, interactions between these species were not more consistent across space. Thus specialized interactions (least generalized) might not actually be between specialist species *per se*, but simply between species with few local interaction partners which might change across sites. Network species richness neither had any significant effect on the turnover of pairwise interactions. Instead, complex synergistic effects of different community and landscape properties will have to be included in order to discover more deeply the mechanisms behind the detailed patterns of pairwise interactions.

High levels of endemism and extremely narrow distributional ranges of some species [42] make *campos rupestres* a unique but also fragile habitat. *Campos rupestres* are under threat from several human activities such as mining, cattle ranching, wood extraction, cultivation, and road construction [43]. Here we confirm the high heterogeneity of species composition across *campo rupestre* habitat and further show an equally high turnover of plant-pollinator interactions. While plants in *campos rupestres* are spatially constrained and distributed in local patches, pollinators disperse freely between these patches. Pollinators could thus function as spatial couplers of otherwise disjunct plant populations and be very important to gene flow between local plant populations; a subject worthy of further study. These findings indicate that conservation management in *campos rupestres* will likely need to consider the protection of a large network of reserves, i.e. a metanetwork [7], in order to maximize representation of species and processes [6].

Future work on interactions could focus on the turnover of functional groups instead of taxonomic species. Perhaps species traits are better predictors of pairwise interactions than actual species identities (e.g. [53]). Trait information could increase our knowledge on which interactions are the most consistent and why, and reveal which, if any, interactions are truly obligatory [28], [36].

We have quantified the beta diversity of interactions across space and investigated the turnover of each pairwise interaction. Beta diversity of interactions is generally high and the identity of pairwise interactions is highly variable across space. A large part of the pairwise interactions constituting plant-pollinator networks seems to be partly random encounters and/or opportunistic interactions whose identity is largely determined by local species abundances. However, pairwise interactions that are locally frequent will tend to be consistent across space if no temporal or spatial constraints are imposed on the species. Thus, beneath the large variation and diversity across space, some species form interactions that are more consistent and predictable. Such interactions represent cornerstones of interacting communities and deserve special attention from ecologists and conservation planners alike.

## Supporting Information

File S1 **Site information.** Altitude, species richness of plants and pollinators, richness of interactions, and the distance to nearest site for the seven sites and for the region in total.
(DOCX)

File S2 **Sampling effort.** Description of sampling effort, including Chao 2 estimator calculation and rarefaction curves.
(DOCX)

File S3 **Species beta diversity between sites.** Detailed values of plant and pollinator beta diversity across all 21 site-pairs.
(DOCX)
