# Correction: Beta Diversity of Plant-Pollinator Networks and the Spatial Turnover of Pairwise Interactions

**DOI:** 10.1371/journal.pone.0117763

**Published:** 2015-02-03

**Authors:** The PLOS ONE Staff

In the Funding section, grant number 2014/01594-4 from the funder São Paulo Research Foundation to DWC is missing. The complete, correct funding information is as follows: São Paulo Research Foundation grant # 2010/51307-0 (FAPESP–VALE), grant # 2011/22635-2, # 2013/05920-0, and 2014/01594-4 to DWC. UNESP grant to MS. Research Productivity fellowship CNPq to LPCM. The funders had no role in study design, data collection and analysis, decision to publish, or preparation of the manuscript.
