# Supplementary material for: Beta Diversity of Plant-Pollinator Networks and the Spatial Turnover of Pairwise Interactions
Source: PLoS One. 2014 Nov 10;9(11):e112903. doi: 10.1371/journal.pone.0112903 (PMC4226610; doi:10.1371/journal.pone.0112903)

**S1.Site information**

Table S1. Altitude, species richness of plants and pollinators, richness of interactions, and the distance to nearest site for the seven sites and for the region in total. All sites are *campo rupestre* habitat with similar physiognomy.

| **Site** | **GPS** | **Altitude (m)** | **#Plants** | **#Pollinators** | **#Interactions** | **Dist. to nearest site (km)** |
| --- | --- | --- | --- | --- | --- | --- |
| Gigante | 19°14'50.31"S - 43°30'36.71"W | 1269 | 31 | 71 | 159 | 4.9 |
| Paulino | 19°15'29.12"S - 43°35'1.93"W | 1073 | 26 | 85 | 175 | 1.7 |
| Tinkerbell | 19°13'14.61"S - 43°34'58.67"W | 1188 | 22 | 59 | 91 | 1.4 |
| Midway | 19°16'13.07"S - 43°33'1.27"W | 1205 | 25 | 53 | 122 | 2.6 |
| Cedro | 19°13'55.48"S - 43°34'35.02"W | 1121 | 27 | 69 | 135 | 1.4 |
| Elefante | 19°17'36.43"S - 43°33'19.92"W | 1181 | 33 | 76 | 159 | 2.6 |
| Soizig | 19°16'22.09"S - 43°34'47.41"W | 1095 | 23 | 68 | 115 | 1.7 |
| **Region** | - | - | **101** | **199** | **768** | - |

Figure S1. Landscape picture of *campos rupestres*  (rupestrian fields). Photo credit: Daniel W. Carstensen.


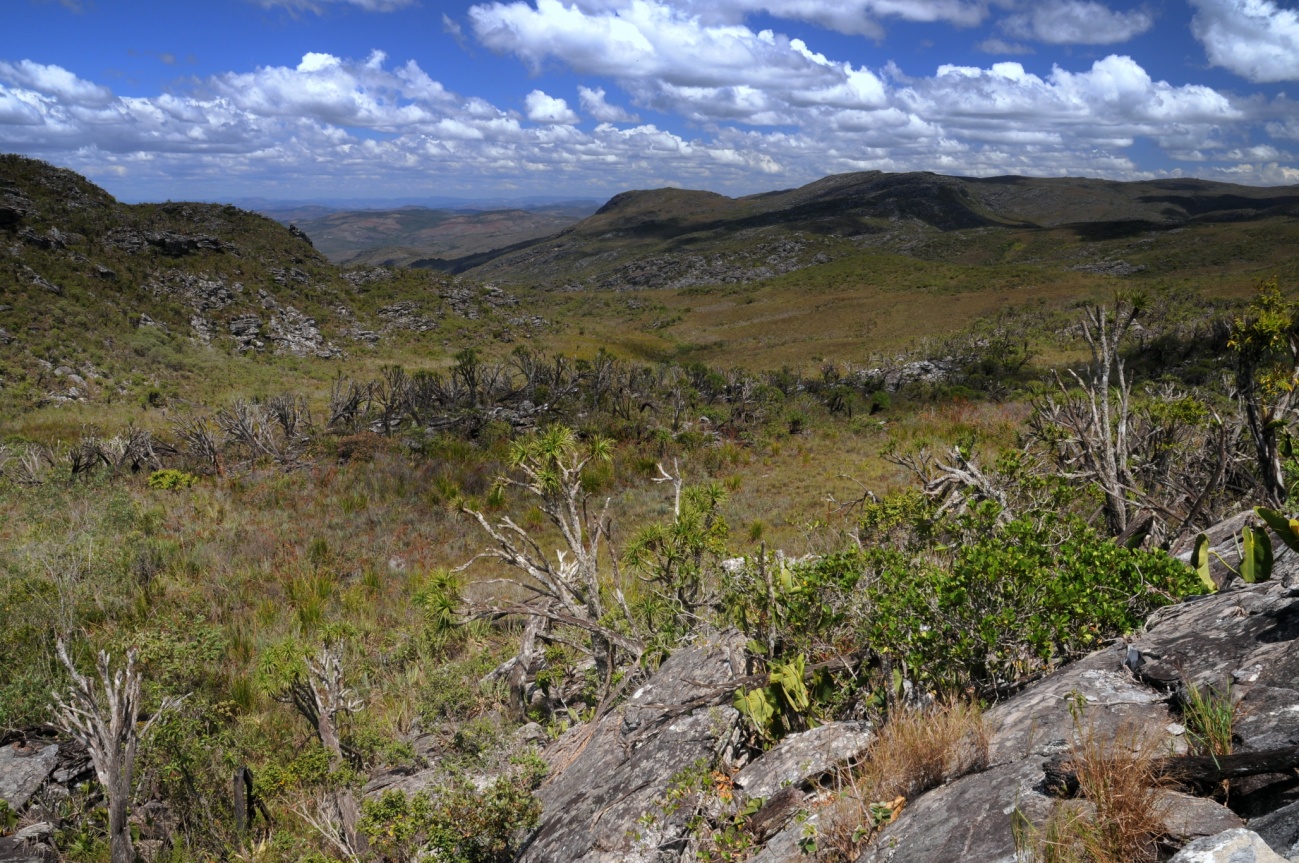

Supplement: File S1 — Site information. Altitude, species richness of plants and pollinators, richness of interactions, and the distance to nearest site for the seven sites and for the region in total. (DOCX) [file pone.0112903.s001.docx]
