# Supplementary material for: Beta Diversity of Plant-Pollinator Networks and the Spatial Turnover of Pairwise Interactions
Source: PLoS One. 2014 Nov 10;9(11):e112903. doi: 10.1371/journal.pone.0112903 (PMC4226610; doi:10.1371/journal.pone.0112903)
Supplement: File S2 — Sampling effort. Description of sampling effort, including Chao 2 estimator calculation and rarefaction curves. (DOCX) [file pone.0112903.s002.docx]

**S2. Sampling effort**

All sites were sampled intensively with equal sampling effort (36±1h per site). It is extremely difficult to reach a rarefaction asymptote for interactions (Chacoff et al. 2012). In order to evaluate the sampling completeness of interactions across our sites in more detail, we used the method of Chao (1987), calculating the Chao 2 estimator of asymptotic species richness (using interactions instead of species):

S_E_ =S_O_ + (L^2^/2M),

where L is the number of interactions that occur in only one sample, M is the number of interactions that occur in exactly two samples, and S_0_ is the observed number of unique interactions. Then, to estimate the percent asymptotic richness detected we calculated the ratio of S_O_ to S_E_ (Chacoff et al. 2012). The average percent asymptotic richness detected across sites was 58.13%, SD = 4.63 (55% detected by Chacoff et al. 2012 for comparison). Based on these calculations and visual inspection of the rarefaction curves, we argue that sites are adequately and, importantly for this study, equally well, sampled.


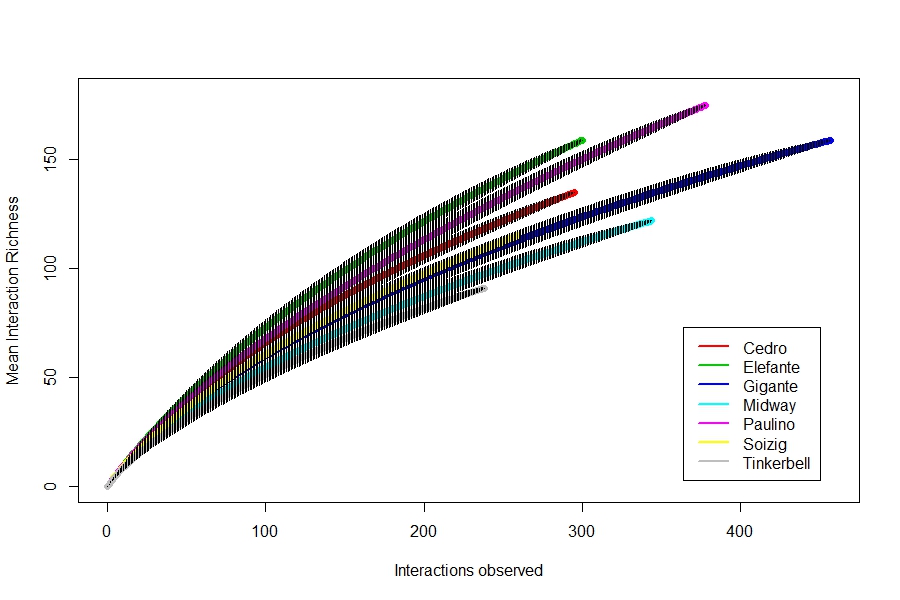


| **Site** | **Chao2** | **Sampling time (min)** |
| --- | --- | --- |
| Gigante | 62.96 | 2190 |
| Paulino | 54.55 | 2220 |
| Tinkerbell | 64.56 | 2100 |
| Midway | 61.30 | 2190 |
| Cedro | 53.45 | 2130 |
| Elefante | 55.19 | 2130 |
| Soizig | 54.89 | 2190 |
| **Average** | **58.13** | **2164** |

Chao A (1987) Estimating the population size capture-recapture data with unequal catchability. Biometrics 43: 783-791.

Chacoff NP, Vázquez DP, Lomáscolo SB, Stevani EL, Dorado J, et al. (2012) Evaluating sampling completeness in a desert plant-pollinator network. Journal of Animal Ecology 81:190-200.
