# Supplementary material for: Beta Diversity of Plant-Pollinator Networks and the Spatial Turnover of Pairwise Interactions
Source: PLoS One. 2014 Nov 10;9(11):e112903. doi: 10.1371/journal.pone.0112903 (PMC4226610; doi:10.1371/journal.pone.0112903)

**S3. Species beta diversity between sites.**

Table S3. Species beta diversity between each of the 21 site-pair combinations. Cells in grey contain values for plant species. Cells in white contain values for pollinator species.

| **Turnover** | Cedro | Elefante | Gigante | Midway | Paulino | Soizig | Tinkerbell |
| --- | --- | --- | --- | --- | --- | --- | --- |
| Cedro | - | 0.53 | 0.86 | 0.58 | 0.62 | 0.60 | 0.63 |
| Elefante | 0.53 | - | 0.88 | 0.62 | 0.53 | 0.46 | 0.60 |
| Gigante | 0.59 | 0.61 | - | 0.79 | 0.93 | 0.93 | 0.85 |
| Midway | 0.52 | 0.57 | 0.55 | - | 0.65 | 0.63 | 0.62 |
| Paulino | 0.49 | 0.55 | 0.69 | 0.64 | - | 0.43 | 0.58 |
| Soizig | 0.43 | 0.47 | 0.57 | 0.59 | 0.52 | - | 0.60 |
| Tinkerbell | 0.55 | 0.59 | 0.62 | 0.57 | 0.63 | 0.46 | - |

Figure S3. Relationship between beta diversity of plants (β_Plants_) and pollinators (β_Pollinators_) across all sites. They are positively correlated (p = 0.008, R^2^ = 0.32), with a larger turnover for plants than for pollinators across sites (α = 0.24). The blue broken line shows the 1:1 relationship.


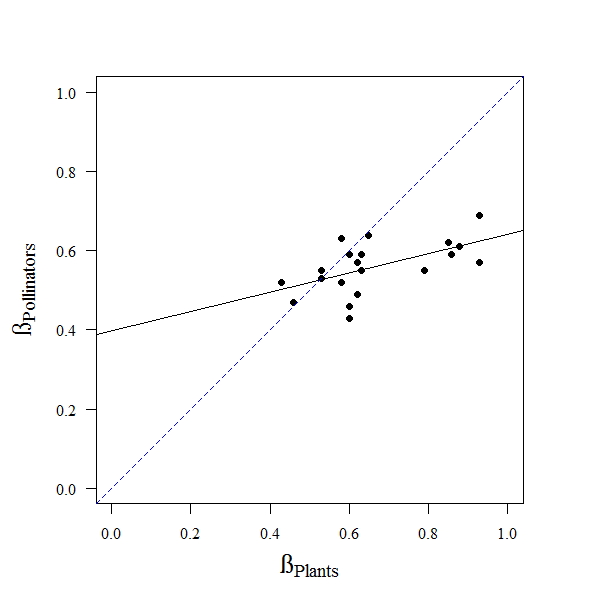

Supplement: File S3 — Species beta diversity between sites. Detailed values of plant and pollinator beta diversity across all 21 site-pairs. (DOCX) [file pone.0112903.s003.docx]
